# Association of Opioid Dose Reduction With Opioid Overdose and Opioid Use Disorder Among Patients Receiving High-Dose, Long-term Opioid Therapy in North Carolina

**DOI:** 10.1001/jamanetworkopen.2022.9191

**Published:** 2022-04-27

**Authors:** Bethany L. DiPrete, Shabbar I. Ranapurwala, Courtney N. Maierhofer, Naoko Fulcher, Paul R. Chelminski, Christopher L. Ringwalt, Timothy J. Ives, Nabarun Dasgupta, Vivian F. Go, Brian W. Pence

**Affiliations:** 1 Gillings School of Global Public Health, Department of Epidemiology, University of North Carolina at Chapel Hill, Chapel Hill; 2 Injury Prevention Research Center, University of North Carolina at Chapel Hill, Chapel Hill; 3 School of Medicine, Department of Medicine, University of North Carolina at Chapel Hill, Chapel Hill; 4 Gillings School of Global Public Health, Department of Health Behavior, University of North Carolina at Chapel Hill, Chapel Hill; 5 Eshelman School of Pharmacy, Division of Practice Advancement and Clinical Education, University of North Carolina at Chapel Hill, Chapel Hill

## Abstract

**Importance:**

Rapid reduction or discontinuation of long-term opioid therapy may increase risk of opioid overdose or opioid use disorder (OUD). Current guidelines for chronic pain management caution against rapid dose reduction but are based on limited evidence.

**Objective:**

To characterize the association between rapid reduction or abrupt discontinuation of opioid therapy (vs maintained or gradual reduction) and incidence of opioid overdose and OUD among patients prescribed high-dose, long-term opioid therapy (HDLTOT).

**Design, Setting, and Participants:**

This retrospective cohort study was conducted among patients aged 18 to 64 years who were prescribed HDLTOT (≥90 daily morphine milligram equivalents for ≥90% of 90 days) from January 2006 to September 2018, with follow-up up to 4 years after cohort entry. Claims data were drawn from a large private health insurer in North Carolina and analyzed from March 1, 2006, to September 30, 2018.

**Exposures:**

Time-varying exposure of rapid dose reduction or discontinuation (>10% dose reduction/week) vs maintenance, increase, or gradual reduction or discontinuation.

**Main Outcomes and Measures:**

The main outcome was incident opioid overdose (fatal or nonfatal) or diagnosed OUD. Inverse probability-weighted cumulative incidence of outcomes were estimated using the cumulative incidence function and hazard ratios (HRs) using marginal structural Fine-Gray models as a function of rapid dose tapering or discontinuation (vs gradual reduction or discontinuation or maintained or increased), accounting for competing risks.

**Results:**

A total of 19 443 patients (median [IQR] age, 49 [41-55] years; 10 073 [51.8%] men) who received HDLTOT were identified. Rapid reduction or discontinuation was associated with higher risk of fatal and nonfatal overdoses compared with gradual reduction after the first year (year 1: HR, 1.43; 95% CI, 0.94-2.18; years 2-4: HR, 1.95; 95% CI, 1.31-2.90). There was no association between rapid reduction or discontinuation and diagnosed OUD through 2 years of follow-up; however, the hazard of incident OUD among patients exposed to rapid tapering or discontinuation was greater 25 to 48 months after the start of follow-up (HR, 1.28; 95% CI, 1.01-1.63).

**Conclusions and Relevance:**

In this cohort study, rapid dose reduction or discontinuation was associated with increased risk of opioid overdose and OUD during long-term follow-up. These findings reinforce prior concerns about safety of rapid dose reductions for patients receiving HDLTOT and highlight the need for caution when reducing opioid doses.

## Key Points

**Question:**

Is rapid dose decrease or discontinuation among patients receiving high-dose, long-term opioid therapy associated with increased risk of opioid-related harms?

**Findings:**

In a retrospective cohort study of 19 443 privately insured patients who received high-dose, long-term opioid therapy, rapid dose reduction or discontinuation (vs dose maintenance or increase or gradual reduction or discontinuation) was associated with increased risk of opioid overdose over 4 years of follow-up.

**Meaning:**

This cohort study found that opioid dose reduction or discontinuation that exceeded current chronic pain management guidelines was associated with increased risk of opioid-related harms, highlighting the importance of caution when reducing opioid doses in order to improve patient safety.

## Teaser

This cohort study assesses the association of rapid reduction or discontinuation of opioid therapy with overdose and opioid use disorder (OUD) among patients receiving high-dose, long-term opioid therapy in North Carolina.

## Introduction

Approximately 20 years into the opioid epidemic in the United States, optimal strategies for long-term opioid therapy (LTOT) for chronic pain remain poorly defined.^1,2^ The clinical need for pain management tools for patients with chronic pain is undisputed; the human toll of widespread opioid prescribing in terms of opioid misuse, opioid use disorder (OUD), and overdoses is equally clear.^3,4,5,6,7,8^ The need for evidence to inform the balancing of these risks and benefits is urgent.^1,2,9,10,11^

Spurred by the 2016 guidelines from the Centers for Disease Control and Prevention (CDC),^9^ numerous recent legislative and policy actions have sought to regulate opioid prescribing to increase patient safety.^12,13,14^ While often written to rein in high-volume prescribers or regulate first prescriptions for acute or postsurgical pain, these actions have had a general chilling effect, with demonstrated opioid prescription reductions or discontinuations for patients with chronic pain associated with these policies, even when they are not the intended policy targets.^15,16^

For patients with chronic and intractable pain, whether or not to reduce or discontinue LTOT and the optimal approach to do so are clinical management questions of particular importance. Some studies have raised concerns that overly rapid reduction or abrupt discontinuation of LTOT may increase patients’ risk of overdose by leading them to turn to illicit drugs to manage their suddenly uncontrolled pain.^16,17^ The CDC guidelines for chronic pain management caution against rapid dose reduction and recommend decreasing dosage by 10% or less per week.^9,15,18^ However, these recommendations are based on expert opinion derived from a very limited evidence base, as stated in the guidelines themselves.^9,10,11,18^

Accordingly, we sought to characterize incidence of OUD and nonfatal and fatal opioid overdose in a cohort of privately insured patients prescribed high-dose LTOT (HDLTOT), comparing outcomes between patients with stable or guideline-concordant gradual opioid dosage reduction vs those with a rapid dose reduction or abrupt discontinuation of opioid therapy. We hypothesized that rapid dose reduction or discontinuation would increase risk of adverse outcomes compared with maintaining or gradually reducing doses. We further hypothesized that both dose maintenance and gradual reduction or discontinuation would have protective associations against adverse outcomes compared with rapid dose reduction or discontinuation.

## Methods

This cohort study was approved by the institutional review board at the University of North Carolina at Chapel Hill and determined to be exempt from informed consent because data were deidentified. This study followed the Strengthening the Reporting of Observational Studies in Epidemiology (STROBE) reporting guideline.

### Study Data and Population

We conducted a retrospective cohort study using deidentified insurance claims from a large private health insurer, covering about one-fifth of North Carolina residents, between January 1, 2006, and September 30, 2018. Included individuals were adults (ages 18-64 years) who received HDLTOT, defined as at least 90 daily morphine milligram equivalents (MME) for at least 90% of 90 consecutive days.^19,20^

We calculated daily MME similarly to definition 2 from Dasgupta et al^21^ (eMethods in the Supplement). Briefly, dose per unit and number of units dispensed for each prescription were multiplied, then divided by days’ supply from the outpatient pharmaceutical claim. This daily dose was then multiplied by an MME conversion factor from CDC tables.^22^ Finally, daily MME was calculated as the sum of MME per day across all prescriptions each day. Overlapping prescriptions for 7 or fewer days were staggered, while those overlapping more than 7 days were assumed to truly overlap.^23^

Patients with a history (using all-available data for lookback^24,25^) of opioid overdose or OUD were excluded, identified using *International Classification of Diseases, Ninth Revision, Clinical Modification *(*ICD-9-CM*), or *International Statistical Classification of Diseases, Tenth Revision, Clinical Modification *(*ICD-10-CM*) codes in insurance claims (eTable 1 in the Supplement). To identify fatal overdoses, claims data were linked to vital records (deaths) from the North Carolina Department of Health and Human Services Division of Public Health using a hierarchical matching algorithm (eFigure 1 in the Supplement).

Patients were followed from the first day after the 90-day HDLTOT classification period until the death, disenrollment, administrative censoring (September 30, 2018), or end of 48 months, whichever came first (eFigure 2 in the Supplement). Patients could reenter the analytic cohort after disenrollment, with follow-up time reset to 0, if they reentered the insurance pool and again met eligibility criteria.

### Exposure

We assessed exposure status at each month of follow-up. During each 30-day period, we compared mean dose during the current month to both the previous month’s mean dose and 6-month rolling mean to classify patients’ prescription trajectories as dose maintained, increased, gradually decreased, rapidly decreased, gradually discontinued, or rapidly discontinued. Comparison to a 6-month rolling mean was included to minimize impacts of short-term dose variabilities on exposure classification. We defined gradual dose reduction following CDC guideline recommendations of no more than 10% dose reduction per week (≤34% per month) and anything faster as rapid dose reduction (eMethods and eTable 2 in the Supplement).^9^

Our primary analyses applied a time-varying dichotomous exposure of rapid decrease or discontinuation vs maintenance or increase or gradual reduction or discontinuation. We used a time-varying intent-to-treat approach, classifying patients as ever exposed to any rapid reduction or discontinuation after their first identified rapid reduction or discontinuation event, vs never exposed.

To address our secondary hypothesis, we used a 3-level time-varying exposure, classifying patients as having had their dosage (1) consistently maintained or increased, (2) ever gradually but never rapidly reduced or discontinued, or (3) ever rapidly reduced or discontinued.

### Outcomes

We examined 4 coprimary outcomes of interest: (1) fatal opioid overdose, identified using *ICD-10* codes from underlying and contributing causes of death in linked death records (eTable 3 in the Supplement), (2) incident nonfatal opioid overdose identified using *ICD-9-CM* and *ICD-10-CM* diagnosis codes from insurance claims (eTable 1 in the Supplement), (3) a combined outcome of incident nonfatal or fatal opioid overdose, and (4) incident OUD identified using diagnosis codes from insurance claims (eTable 1 in the Supplement). Death (all-cause) was treated as a competing risk^26,27^ for incident nonfatal overdose and incident OUD, as was death not attributed to opioid overdose for incident fatal overdose and incident overdose (fatal or nonfatal).

### Patient Characteristics

Time-fixed patient characteristics at the index date were sex and history of opioid use prior to the 90-day HDLTOT classification period. All time-updated patient characteristics were identified prior to the start of each 30-day exposure window to ensure correct temporal ordering (eFigure 2 in the Supplement). Time-updated demographic characteristics included age (modeled as quadratic) and calendar year (categorical to avoid small cell counts: 2006-2010, 2011-2012, 2013-2014, and 2015-2018, based on functional form analysis and accounting for waves of the opioid epidemic^28^ and changing policies) at the start of the prior 30-day window. Time-updated 5-digit zip-code level characteristics (missing for 22 individuals excluded from the analytic cohort) included percentage of individuals in the zip code identifying as Black and percentage identifying as other race, including American Indian and Alaska Native, Asian, Native Hawaiian and other Pacific Islander, or individuals who identify as another race not listed or 2 or more races (both categorized based upon quartiles), both obtained from the American Community Survey (ACS),^29^ and rural-urban commuting area (RUCA) codes applied to the zip code^30^ (categorized as metropolitan, micropolitan, and small town/rural) at the start of the prior 30-day window. Zip code–level characteristics, including race, were merged with patient zip code from insurance member files and were included in propensity score models to account for community level and geographic differences that may be associated with opioid prescribing (exposure) and opioid-related harms (outcome). Time-updated diagnoses of depression, anxiety, posttraumatic stress disorder, substance use disorder other than OUD (eg, alcohol use disorder), and cancer were identified using an all-available lookback prior to the start of the previous 30-day period. Time-varying prescriptions included selective serotonin reuptake inhibitors, non–selective serotonin reuptake inhibitor antidepressants (eg, bupropion, trazodone), benzodiazepines, other anxiolytics (eg, buspirone), naloxone, and whether the patient received any extended-release opioids during the previous 30-day period. Time-varying derived indications included diagnosed acute pain, chronic pain, or invasive surgery in the 6-month period before the start of the previous 30-day period.

### Statistical Analysis

We first calculated median change in dose by exposure status between baseline to month 12 and baseline to month 48. To estimate the association between rapid opioid dose reduction or discontinuation with time-to-incident opioid overdose or diagnosed OUD, we related exposure status through month *t* to outcome occurrence during month *t* + 1, implemented with inverse probability (IP) weighted survival curves and marginal structural models.^31,32^ We used stabilized IP treatment weights (IPTW) to account for time-dependent confounding^33^ (eMethods in the Supplement). To address possible selection bias stemming from potentially informative censoring, we calculated stabilized IP censoring weights (IPCW). We then multiplied IPCW by IPTW to obtain IPTC-weights (IPTCW).

We estimated crude and weighted cumulative incidence of (1) fatal opioid overdose, (2) nonfatal opioid overdose, (3) nonfatal or fatal opioid overdose, and (4) incident OUD using the cumulative incidence function through 48 months of follow-up, accounting for competing risks.^34,35^ We calculated risk differences at multiple time points, obtaining 95% CI using robust variance estimators to account for repeated observations.

We used weighted Fine-Gray models to estimate subdistribution hazard ratios (HRs), accounting for competing risks.^34^ We used an infinitesimal jackknife^36^ to compute robust SEs and Efron method^37^ to handle tied event times. We assessed the proportional hazards assumption using Schoenfeld residuals, with models stratified by follow-up time, where appropriate, to handle violations.

We conducted additional sensitivity analyses (eMethods in the Supplement). First, to examine impacts of baseline opioid dose variability on cohort selection, we restricted the analytic cohort to patients determined to have stable baseline dosing. Second, to address potential outcome misclassification, we examined only nonfatal overdoses occurring during an emergency department or inpatient event.

We used SAS version 9.4 (SAS Institute) for data management and R version 3.6.0 (R Project for Statistical Computing) for analyses (eTable 3 in the Supplement). Significance was defined as 95% CIs that did not cross 0 for risk differences or that did not cross 1 for HRs. Data were analyzed from March 1, 2006, to September 30, 2018.

## Results

We identified 19 443 patients who received HDLTOT. Median (IQR) age at day 0 was 49 (41-55) years and 10 073 (51.8%) were men (Table 1). Most patients had prior opioid exposure (11 588 patients [59.6%]). In the 6 months before study follow-up, 17 317 patients (89.1%) had a chronic pain diagnosis. One-third of patients had ever been diagnosed with depression (6399 patients [32.9%]) or anxiety (6427 patients [33.1%]), and 2694 patients (13.9%) had a history of cancer.

**Table 1.** Characteristics at Baseline of 19 443 Patients Receiving High-Dose, Long-term Opioid Therapy in North Carolina, 2006-2018

Characteristic	Participants, No. (%) (N = 19 443)
Age, median (IQR), y	49 (41-55)
Sex	
Women	9313 (48.2)
Men	10 073 (51.8)
Calendar year	
2006	2915 (15.0)
2007	1498 (7.7)
2008	1492 (7.7)
2009	1454 (7.5)
2010	1367 (7.0)
2011	1183 (6.1)
2012	1194 (6.1)
2013	1228 (6.3)
2014	2194 (11.3)
2015	1790 (9.2)
2016	1236 (6.4)
2017	1427 (7.3)
2018	465 (2.4)
Prior opioid exposure, ever	11 588 (59.6)
Diagnosis	
Cancer	2694 (13.9)
Depression	6399 (32.9)
Anxiety	6427 (33.1)
PTSD	420 (2.2)
SUD	1782 (9.2)
Pain diagnosis, past 6 mo	
Acute	4926 (25.3)
Chronic	17 317 (89.1)
Surgery, past 6 mo	2371 (12.2)
Medication use, past mo	
Benzodiazepine	7873 (40.5)
SSRI	3984 (20.5)
Anxiolytic	641 (3.3)
Antidepressant	5794 (29.8)
Naloxone	21 (0.1)
ER/LA	11 360 (58.4)
Log cumulative MME, median (IQR)	9.64 (9.39-9.98)

Abbreviations: ER/LA, extended-release/long-acting opioid; MME, morphine milligram equivalent; PTSD, posttraumatic stress disorder; SSRI, selective serotonin reuptake inhibitor; SUD, substance use disorder.

During follow-up, there were 59 fatal opioid overdoses, 215 nonfatal overdoses, 268 fatal or nonfatal overdoses (if individuals experienced a nonfatal overdose before a fatal overdose, only the first [nonfatal] overdose was considered for the combined outcome), and 2796 incident OUD diagnoses (Table 2). Across the 4 outcomes, median follow-up ranged from 15 to 17 months, and nearly half (46%-49%) of follow-up time was classified as exposed to rapid reduction or discontinuation. Competing risk of death was observed among 4.6% to 4.8% of patients. Among patients exposed to rapid dose decrease or discontinuation by month 12, median (IQR) dose change was −49.7% (−91.2% to −5.5%) from baseline to month 12 and −54.1% (−100.0% to 2.5%) by month 48 among those exposed by month 48. Among unexposed patients, median (IQR) dose change was 3.7% (−2.6% to 33.3%) by month 12 and 23.0% (0% to 72.9%) by month 48.

**Table 2.** Incident Fatal Opioid Overdose, Nonfatal Opioid Overdose, Fatal or Nonfatal Opioid Overdose, and Opioid Use Disorder Overall and by Exposure Status Among Patients Receiving High-Dose, Long-term Opioid Therapy in North Carolina, 2006-2018

Outcome, follow-up mo	No.					
	Overall		Maintained, increased, or gradually reduced or discontinued		Rapidly reduced or discontinued	
	Events	Follow-up, person-months^a^	Events	Follow-up, person-months	Events	Follow-up, person-months
Opioid overdose						
Fatal	59	475 959	26	244 696	33	231 263
0-12	29	205 482	17	148 420	12	57 061
13-48	30	270 477	9	96 275	21	174 202
Nonfatal	215	472 603	93	244 106	122	228 497
0-12	99	204 929	64	148 181	35	56 747
13-48	116	267 674	29	95 924	87	171 750
Fatal or nonfatal^b^	268	472 604	115	244 106	153	228 497
0-12	126	204 929	79	148 181	47	56 747
13-48	142	267 674	36	95 924	106	171 750
Opioid use disorder	2796	432 004	1603	233 382	1193	198 622
0-12	1534	197 116	1124	144 382	410	52 734
13-24	703	113 981	326	52 287	377	61 694
25-48	559	120 907	153	36 713	406	84 194

^a^ Person-months of follow-up differ across each outcome analysis because an individual may have experienced a nonfatal outcome (eg, opioid use disorder or nonfatal opioid overdose) prior to a fatal overdose. Therefore, that individual would contribute fewer person-months to the analysis with the nonfatal outcome than to the fatal opioid overdose outcome analysis.

^b^ Some individuals had both a nonfatal and then a fatal overdose; thus the number of combined events is less than the number of fatal overdoses plus the number of nonfatal overdoses.

**Figure 1.**
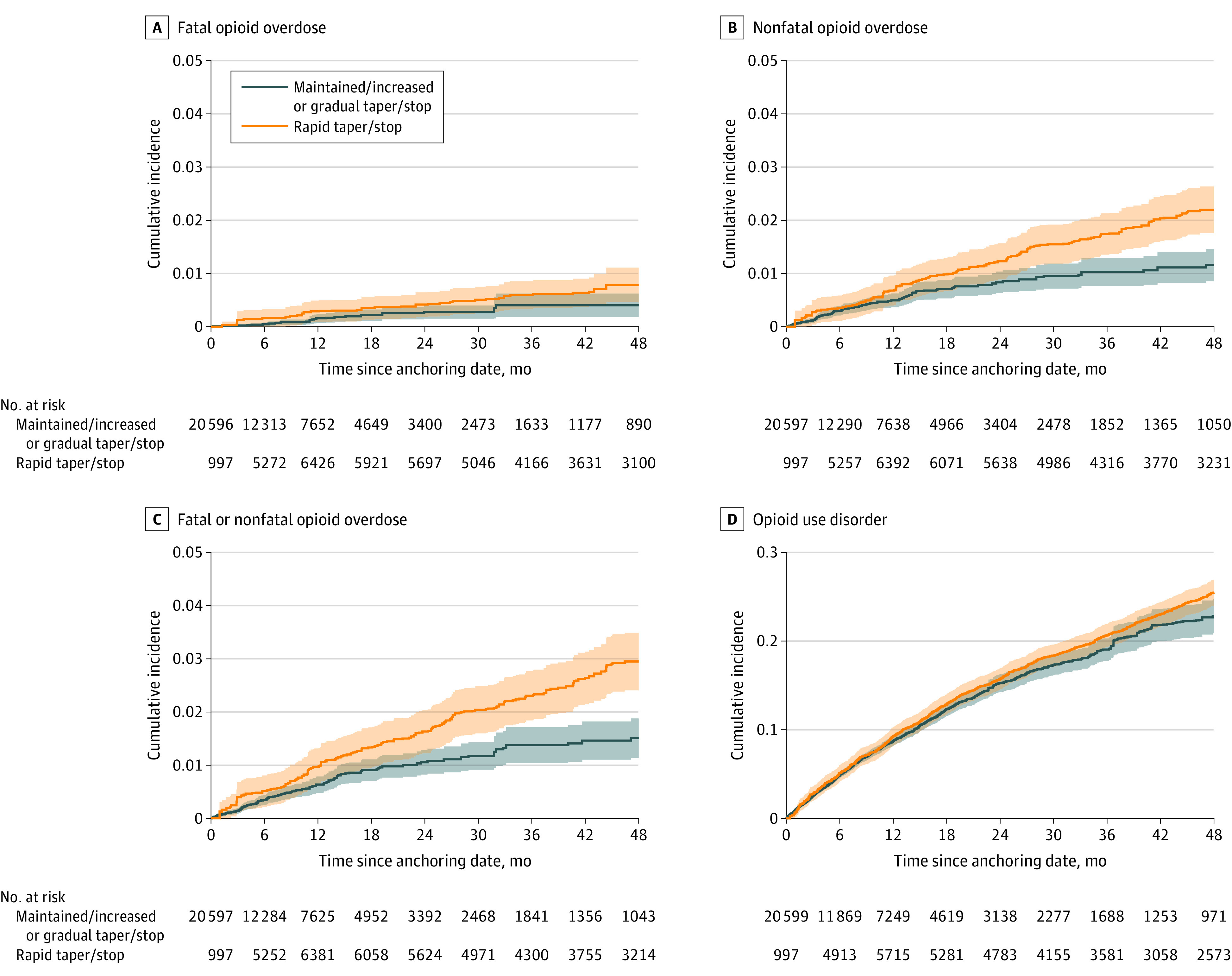
Inverse Probability of Treatment and Censoring–Weighted Cumulative Incidence Curves by Primary Exposure Status Among 19 443 patients receiving high-dose, long-term opioid therapy in North Carolina from 2006 to 2018. Shading indicates 95% CI.

Crude (eFigure 3 in the Supplement) and weighted (Figure 1A-C) cumulative incidences of fatal opioid overdose, nonfatal opioid overdose, and combined fatal or nonfatal opioid overdose were consistently higher for patients exposed to rapid dose reduction or discontinuation compared with patients with maintained, increased, or gradually reduced or discontinued dosage (eTable 5 in the Supplement). We found no notable difference in incident OUD across exposure groups during the first 12 months of follow-up (risk difference, 0.53%; 95% CI, −0.65 to 1.71), after which the weighted cumulative incidence of OUD was higher among patients ever exposed to rapid dose reduction or discontinuation, although with considerable confidence interval overlap (Figure 1D). Differences in cumulative incidence were more pronounced after 2 years of follow-up for all 4 outcomes examined, with the largest difference between cumulative incidence curves toward the end of the follow-up period. Specifically, the weighted risk difference of the combined outcome of fatal or nonfatal opioid overdose among patients who ever experienced rapid dose reduction or discontinuation of opioid therapy, compared with patients with maintained, increased, or gradually reduced or discontinued dosage, was 0.25% (95% CI −0.04 to 0.54) at 3 months of follow-up and 0.58% (95% CI, 0.11 to 1.04) at 2 years of follow-up (eTable 5 in the Supplement).

**Figure 2.**
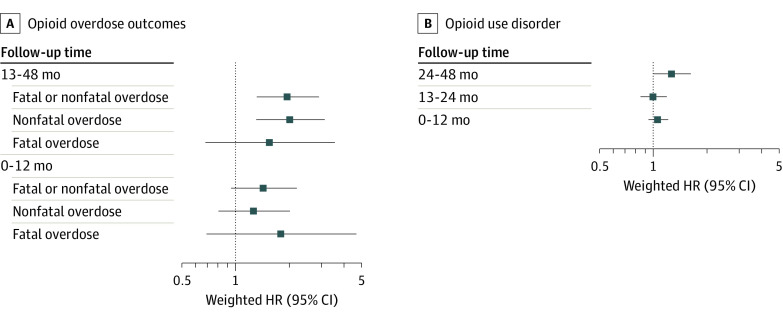
Inverse Probability of Treatment and Censoring–Weighted Hazard Ratios (HRs) Comparing Patients Exposed to Rapid Tapering or Discontinuation vs Those Who Had Their Dosage Maintained Among 19 443 patients receiving high-dose, long-term opioid therapy or gradually tapered or discontinued in North Carolina from 2006 to 2018.

Tests indicated that the proportional hazards assumption was not upheld, indicating separate estimates for months 1 to 12 vs 13 to 48 of follow-up for overdose outcomes and months 1 to 12, 13 to 24, and 25 to 48 of follow-up for OUD. Among patients ever exposed to rapid dose reduction or discontinuation, compared with those never exposed, the weighted hazard of incident nonfatal or fatal opioid overdose was increased with time (year 1: weighted HR, 1.43; 95% CI, 0.94 to 2.18; years 2-4: weighted HR, 1.95; 95% CI, 1.31 to 2.90) (Figure 2; eTable 6 in the Supplement). A similar trend was observed for each of these 2 outcomes alone. The hazard of incident OUD comparing patients ever exposed to rapid reduction or discontinuation vs those never exposed was not significantly higher through 2 years of follow-up (year 1: weighted HR, 1.07; 95% CI, 0.94 to 1.21; year 2: weighted HR, 1.01; 95% CI, 0.85 to 1.19). However, the hazard of incident OUD among patients exposed to rapid reduction or discontinuation was notably higher 25 to 48 months after the start of follow-up (weighted HR, 1.28; 95% CI, 1.01 to 1.63).

**Figure 3.**
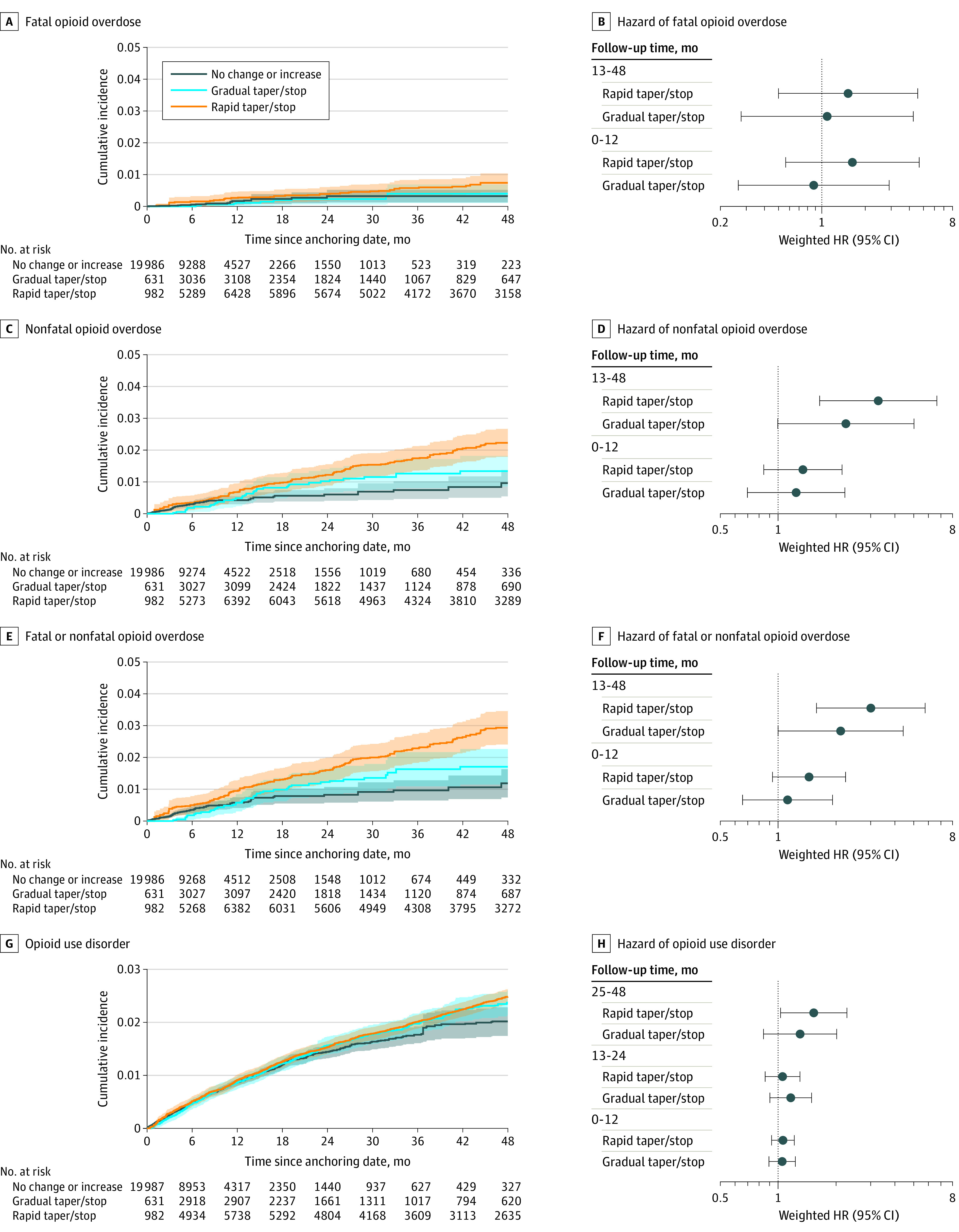
Inverse Probability of Treatment and Censoring–Weighted Cumulative Incidence Curves and Hazard Ratios (HRs) by Exposure Status Using a 3-Level Exposure Coding Among 19 443 patients receiving high-dose, long-term opioid therapy in North Carolina from 2006 to 2018. Shading indicates 95% CI.

When using a 3-category exposure, patients exposed to rapid dose reduction or discontinuation were at consistently higher risk of fatal or nonfatal opioid overdose than patients with maintained or increased dosage (Figure 3A-C). For the first 6 to 9 months of follow-up, patients with gradual dose reduction or discontinuation had the lowest risk of all outcomes. After the first year of follow-up, we observed a dose-response association between dose trajectory and risk of fatal opioid overdose or nonfatal opioid overdose (Figure 3A-F). Within 2 to 4 years after the start of follow-up, patients exposed to any dose reduction or discontinuation had higher risk of incident OUD than those never exposed (gradual: HR, 1.30; 95% CI, 0.84 to 2.01; rapid: 1.52; 95% CI, 1.03 to 2.26), without evidence of a dose-response association (Figure 3D; eTable 7 in the Supplement). Sensitivity analyses using a subsample of patients with stable baseline dosing (eTables 8-10 in the Supplement) and of the nonfatal opioid overdose definition (eTables 11-13 in the Supplement) resulted in similar trends as seen in primary analyses.

## Discussion

In this cohort study of privately insured patients in North Carolina with 12 years of data, we characterized incidence of fatal and nonfatal opioid overdoses and OUD among patients receiving HDLTOT whose dosages were reduced or discontinued more rapidly than recommended by CDC guidelines compared with patients whose opioid therapy was either maintained or gradually reduced or discontinued in a manner consistent with guidelines. Rapid reduction or discontinuation was associated with higher risk of opioid overdoses after the first year of follow-up, and the risk increased with longer follow-up time. When considered separately, those with gradual reduction or discontinuation had the lowest incidence of adverse outcomes during the first 6 to 9 months of follow-up; as follow-up progressed, those without dosage decreases had the lowest incidence, with rapid reduction or discontinuation demonstrating the highest incidence for all overdose outcomes and gradual reduction or discontinuation an intermediate incidence. OUD incidence did not differ between gradually and rapidly reduced patients and was considerably higher during 2 to 4 years of follow-up than among those who received a maintained or increasing opioid dose.

A 2021 study by Agnoli et al^38^ similarly found an association of opioid dose reduction rapidity with nonfatal opioid overdoses, although it did not examine fatal overdoses or OUD. Other studies have reported that opioid discontinuation was associated with increased overdose mortality,^39,40^ emergency department visits,^41^ and heroin use.^17^ We followed patients up to 4 years, thereby assessing the incidence of opioid-related harm over time in greater detail, and were able to examine both fatal and nonfatal overdoses as well as OUD. We also used a stringent definition to determine stable opioid prescribing, consistent with current CDC guidelines (≤10% change per week). Our study, along with prior studies, affirms the potential harms of rapid opioid dose reduction or discontinuation. Such findings have great importance for current policy and practice, as evidenced by recent CDC guidance warning against misapplication of CDC guidelines.^18,42^

When examining guideline-concordant gradual dose reduction separately, we found that gradual reductions had a protective association compared with maintained HDLTOT for 6 to 9 months. However, these associations disappeared after more than a year of HDLTOT, at which point even gradual reduction appeared to increase risk of adverse outcomes compared with sustained HDLTOT, although the increase was less than that for rapid discontinuation. The increased risk associated with gradual dose reduction may be owing to patients’ development of tolerance, after which even gradual reductions may lead to persistent uncontrolled pain,^43^ mental health concerns,^38^ and potential use of diverted or illicit opioids for pain management,^17^ thereby increasing risk of overdoses and OUD. Patients receiving HDLTOT whose medications are reduced or discontinued may feel stigmatized and even experience reduced access to care.^43,44,45^ Development of tolerance, along with the observation that most decreases occurred after 6 months of follow-up, may also help explain the lack of association between rapid dose reduction or discontinuation and opioid-related harms in the first year of follow-up. The long follow-up period in our study facilitates insights into implications for clinical decision-making for patients with HDLTOT.

### Limitations

Our study has several limitations. First, while we developed a directed acyclic graph to control for measured confounding in this study, we could not address potential unmeasured confounding. However, our use of weighted marginal structural models is an important advance in controlling time-varying confounding without blocking causal mediation pathways,^31,46,47^ avoiding bias incurred by standard regression models used in prior studies. Second, we used *ICD-9-CM* and *ICD-10-CM* codes to identify OUD diagnoses, which have low sensitivity and typically underestimate OUD prevalence.^48^ However, this outcome misclassification is likely nondifferential, thereby biasing results toward the null. Similarly, there could be nondifferential underascertainment of nonfatal opioid overdoses in claims data, especially with use of naloxone in the community. Third, our privately insured patient sample may not be representative of patients with Medicaid, Medicare, or no insurance who receive long-term opioids. Fourth, claims data do not provide information on motivation for opioid dosage changes. Fifth, many patients disenrolled before the end of follow-up, and our approach relied on the assumption that IPTCW adequately accounted for informative censoring.

## Conclusions

In this cohort study of privately insured patients receiving HDLTOT, we found that rapid dose reduction or discontinuation, in excess of CDC guidelines, was associated with increased risk of opioid overdose and OUD over 4 years of follow-up. Guideline-concordant gradual reduction or discontinuation had a protective association compared with maintaining or rapidly decreasing doses for the first 9 months of follow-up; however, in the longer term, maintenance of HDLTOT conferred the lowest risk of adverse outcomes compared with rapid decrease, which conferred the highest risk, and gradual decrease, which constituted an intermediate level of risk. These findings reinforce concerns about the safety of precipitous opioid dose reductions for patients receiving HDLTOT and highlight the need for clinicians to monitor patients closely in the long term when reducing opioid doses.

## Notes

Supplement.
eMethods.
**eTable 1.** *ICD-9-CM* and *ICD-10-CM* Diagnostic Codes Used to Identify Opioid Overdose and Opioid Use Disorder (OUD) in Claims Data **eTable 2.** Algorithm Used to Classify Monthly Prescription Trajectories **eTable 3.** *ICD-10* Codes Used to Identify Fatal Opioid Overdose in Linked Death Records **eTable 4.** R Packages Used in Analyses **eTable 5.** IPTCW Risk Differences (95% CI) for the Association Between Rapid Tapering or Discontinuation of Opioid Treatment and Outcomes at Multiple Points of Follow-up **eTable 6.** Unweighted and IPTC-Weighted Hazard Ratios (HR) for Outcomes Comparing Patients Exposed to Rapid Tapering or Discontinuation vs Patients Who Had Their Dosage Maintained or Gradually Tapered or Discontinued **eTable 7.** Unweighted and IPTC-Weighted Hazard Ratios (HR) for Outcomes Comparing Patients Exposed to Rapid Reduction or Discontinuation or Exposed to Gradual Reduction or Discontinuation vs Patients Who Had Their Dosage Maintained or Gradually Tapered or Discontinued **eTable 8.** Outcome Frequency Overall and by Exposure Status Among a Subsample of 12 364 Patients Determined to Have Stable Baseline Dosing **eTable 9.** IPTCW Risk Differences (95% CI) for the Association Between Rapid Tapering or Discontinuation of Opioid Treatment and Outcomes Among a Subsample of 12 364 Patients Determined to Have Stable Baseline Dosing at Multiple Points of Follow-up **eTable 10.** Unweighted and IPTC-Weighted Hazard Ratios (HR) for Outcomes Comparing Patients Exposed to Rapid Tapering or Discontinuation vs Patients Who Had Their Dosage Maintained or Increased or Gradually Tapered or Discontinued Among a Subsample of 12 364 Patients Determined to Have Stable Baseline Dosing **eTable 11.** Outcome Frequency Overall and by Exposure Status, Considering Only Nonfatal Opioid Overdoses Occurring During an Emergency Department or Inpatient Event **eTable 12.** IPTCW Risk Differences (95% CI) for the Association Between Rapid Tapering or Discontinuation of Opioid Treatment and Overdose Outcomes, Considering Only Nonfatal Opioid Overdoses Occurring During an Emergency Department or Inpatient Event **eTable 13.** Unweighted and IPTC-Weighted Hazard Ratios (HR) for Overdose Outcomes Comparing Patients Exposed to Rapid Tapering or Discontinuation vs Patients Who Had Their Dosage Maintained or Gradually Tapered or Discontinued, Considering Only Nonfatal Opioid Overdoses Occurring During an Emergency Department or Inpatient Event **eFigure 1.** Death Match Algorithm **eFigure 2.** Timelines of Eligibility and Exposure Ascertainment and Covariate and Outcome Ascertainment **eFigure 3.** Crude Cumulative Incidence of Outcomes by Primary Exposure Status **eFigure 4.** Crude Cumulative Incidence of Outcomes by Exposure Status Using a Categorical Exposure Definition
eReferences
