# Supplementary material for: Association of Opioid Dose Reduction With Opioid Overdose and Opioid Use Disorder Among Patients Receiving High-Dose, Long-term Opioid Therapy in North Carolina
Source: JAMA Netw Open. 2022 Apr 27;5(4):e229191. doi: 10.1001/jamanetworkopen.2022.9191 (PMC9047650; doi:10.1001/jamanetworkopen.2022.9191)
Supplement: Supplement. — eMethods. eTable 1. ICD-9-CM and ICD-10-CM Diagnostic Codes Used to Identify Opioid Overdose and Opioid Use Disorder (OUD) in Claims Data eTable 2. Algorithm Used to Classify Monthly Prescription Trajectories eTable 3. ICD-10 Codes Used to Identify Fatal Opioid Overdose in Linked Death Records eTable 4. R Packages Used in Analyses eTable 5. IPTCW Risk Differences (95% CI) for the Association Between Rapid Tapering or Discontinuation of Opioid Treatment and Outcomes at Multiple Points of Follow-up eTable 6. Unweighted and IPTC-Weighted Hazard Ratios (HR) for Outcomes Comparing Patients Exposed to Rapid Tapering or Discontinuation vs Patients Who Had Their Dosage Maintained or Gradually Tapered or Discontinued eTable 7. Unweighted and IPTC-Weighted Hazard Ratios (HR) for Outcomes Comparing Patients Exposed to Rapid Reduction or Discontinuation or Exposed to Gradual Reduction or Discontinuation vs Patients Who Had Their Dosage Maintained or Gradually Tapered or Discontinued eTable 8. Outcome Frequency Overall and by Exposure Status Among a Subsample of 12 364 Patients Determined to Have Stable Baseline Dosing eTable 9. IPTCW Risk Differences (95% CI) for the Association Between Rapid Tapering or Discontinuation of Opioid Treatment and Outcomes Among a Subsample of 12 364 Patients Determined to Have Stable Baseline Dosing at Multiple Points of Follow-up eTable 10. Unweighted and IPTC-Weighted Hazard Ratios (HR) for Outcomes Comparing Patients Exposed to Rapid Tapering or Discontinuation vs Patients Who Had Their Dosage Maintained or Increased or Gradually Tapered or Discontinued Among a Subsample of 12 364 Patients Determined to Have Stable Baseline Dosing eTable 11. Outcome Frequency Overall and by Exposure Status, Considering Only Nonfatal Opioid Overdoses Occurring During an Emergency Department or Inpatient Event eTable 12. IPTCW Risk Differences (95% CI) for the Association Between Rapid Tapering or Discontinuation of Opioid Treatment and Overdose Outcomes, Conside [file jamanetwopen-e229191-s001.pdf]

Supplemental Online Content

DiPrete BL, Ranapurwala SI, Maierhofer CN, et al. Association of opioid dose reduction with opioid overdose and opioid use disorder among patients receiving high-dose long-term opioid therapy in North Carolina. *JAMA Netw Open*. 2022;5(4):e229191.
doi:10.1001/jamanetworkopen.2022.9191

eMethods.

eTable 1. *ICD-9-CM* and *ICD-10-CM* Diagnostic Codes Used to Identify Opioid Overdose and Opioid Use Disorder (OUD) in Claims Data

eTable 2. Algorithm Used to Classify Monthly Prescription Trajectories

eTable 3. *ICD-10* Codes Used to Identify Fatal Opioid Overdose in Linked Death Records

eTable 4. R Packages Used in Analyses

eTable 5. IPTCW Risk Differences (95% CI) for the Association Between Rapid Tapering or Discontinuation of Opioid Treatment and Outcomes at Multiple Points of Follow-up

eTable 6. Unweighted and IPTC-Weighted Hazard Ratios (HR) for Outcomes Comparing Patients Exposed to Rapid Tapering or Discontinuation vs Patients Who Had Their Dosage Maintained or Gradually Tapered or Discontinued

eTable 7. Unweighted and IPTC-Weighted Hazard Ratios (HR) for Outcomes Comparing Patients Exposed to Rapid Reduction or Discontinuation or Exposed to Gradual Reduction or Discontinuation vs Patients Who Had Their Dosage Maintained or Gradually Tapered or Discontinued

eTable 8. Outcome Frequency Overall and by Exposure Status Among a Subsample of 12 364 Patients Determined to Have Stable Baseline Dosing

eTable 9. IPTCW Risk Differences (95% CI) for the Association Between Rapid Tapering or Discontinuation of Opioid Treatment and Outcomes Among a Subsample of 12 364 Patients Determined to Have Stable Baseline Dosing at Multiple Points of Follow-up

eTable 10. Unweighted and IPTC-Weighted Hazard Ratios (HR) for Outcomes Comparing Patients Exposed to Rapid Tapering or Discontinuation vs Patients Who Had Their Dosage Maintained or Increased or Gradually Tapered or Discontinued Among a Subsample of 12 364 Patients Determined to Have Stable Baseline Dosing

eTable 11. Outcome Frequency Overall and by Exposure Status, Considering Only Nonfatal Opioid Overdoses Occurring During an Emergency Department or Inpatient Event

eTable 12. IPTCW Risk Differences (95% CI) for the Association Between Rapid Tapering or Discontinuation of Opioid Treatment and Overdose Outcomes, Considering Only Nonfatal Opioid Overdoses Occurring During an Emergency Department or Inpatient Event

© 2022 DiPrete BL et al. *JAMA Network Open.*

eTable 13. Unweighted and IPTC-Weighted Hazard Ratios (HR) for Overdose Outcomes Comparing Patients Exposed to Rapid Tapering or Discontinuation vs Patients Who Had Their Dosage Maintained or Gradually Tapered or Discontinued, Considering Only Nonfatal Opioid Overdoses Occurring During an Emergency Department or Inpatient Event

eFigure 1. Death Match Algorithm

eFigure 2. Timelines of Eligibility and Exposure Ascertainment and Covariate and Outcome Ascertainment

eFigure 3. Crude Cumulative Incidence of Outcomes by Primary Exposure Status

eFigure 4. Crude Cumulative Incidence of Outcomes by Exposure Status Using a Categorical Exposure Definition

eReferences

This supplemental material has been provided by the authors to give readers additional information about their work.

eMethods.

Detailed patient inclusion/exclusion

Patients were excluded if they had a history (using all-available data for lookback[1,2]) of opioid overdose or OUD (n=1,992), identified using International Classification of Diseases (ICD) version 9, clinical modification (ICD-9-CM) or ICD-10-CM codes in insurance claims. Exposure status could not be assessed prior to the end of the first 30-day period; therefore, we excluded 869 patients who were not followed for ≥30 days past the end of the baseline eligibility period. Time-updated five-digit ZIP-code level characteristics were missing for 22 individuals who were excluded from the analytic cohort.

Patients could reenter the analytic cohort after disenrollment, with follow-up time reset to 0, if they reentered the insurance pool and again met eligibility criteria (n=1,867). The statistical approach accounted for repeated measures and variable follow-up duration.

Calculating Daily MME

First, using dose and quantity indicated in the prescription's National Drug Code and prescription days' supply recorded in the outpatient pharmaceutical claim record, dose per unit and number of units dispensed were multiplied and then divided by prescription days' supply, with the resulting milligrams of medication/day then multiplied by an MME conversion factor from CDC conversion tables for each prescription.[3] When overlapping prescriptions of the same ingredient occurred, we assumed that if the start date was ≤7 days before the end date of the previous prescription, this reflected an early refill and the start date of that prescription was pushed forward to the end date of the previous prescription.[4] Otherwise, the prescriptions were assumed to truly overlap. We allowed for gaps in prescriptions in the baseline period by only requiring ≥90% days at ≥90 MMEs. Daily MME for each calendar day was then calculated as the sum of MMEs per day across all prescriptions a patient had on that day.

Defining exposure trajectories

During each 30-day period, we compared average dose during the current month to both (1) average dose from the previous month and (2) a 6-month rolling average to classify patients' prescription trajectories as dose maintained, increased, gradually decreased, rapidly decreased, gradually discontinued, or rapidly discontinued (eTable 2). Specifically, we determined a patient's average dose in the current month and calculated the change in dose from the previous month as well as the change in dose from a 6-month rolling average and used both of these values to determine dose trajectory. We included the comparison with a 6-month rolling average, in addition to accounting for overlapping prescriptions, in order to minimize the impact of short-term dose variabilities on exposure classification. We defined gradual dose reduction following CDC guideline recommendations of ≤10% dose reduction per week (≤34% per month) and anything faster as rapid dose reduction.

Deriving inverse probability weights

We used stabilized IP treatment weights (IPTW) to account for time-dependent confounding.[5] To calculate the IPTW denominator, we used pooled logistic regression to model the probability of experiencing a first rapid dose reduction/discontinuation event during each month of follow-up, given relevant baseline and time-varying confounders. The IPTW denominator included continuous time (restricted cubic spline with 4 knots); all confounders described above; and a one-month lag of other prescriptions, pain (acute or chronic), and surgery. The IPTW numerator was calculated as above, with confounders removed from the model. To account for possible selection bias stemming from potentially informative censoring, we calculated stabilized IP censoring weights (IPCW) in a manner analogous to IPTW, modeling the probability of remaining uncensored given relevant confounders and exposure status. We then multiplied IPCW by IPTW to obtain IPTC-weights.

We examined the weights distribution to ensure a mean of one, and truncated weights at the 0.02nd and 99.98th percentiles to minimize the influence of extreme weights.[5] Covariate balance was assessed using standardized mean differences. After weighting, covariates were well balanced.

© 2022 DiPrete BL et al. *JAMA Network Open.*

Sensitivity Analyses

First, to address potential issues around cohort selection with regard to baseline variability in opioid dosing, we restricted the analytic cohort to patients determined to have “stable” baseline dosing patterns, defined as a change of $\leq$30 MME in the 30-day running average between each 30-day period during the 90-day baseline eligibility period and replicated primary analyses in this sub-sample. Second, to address potential outcome misclassification for coprimary outcomes (2) and (3), we restricted nonfatal overdoses to those occurring during an emergency department (ED) or inpatient event in order to assess the potential impact of using all outpatient claims to identify clinically recognized nonfatal opioid overdoses.

© 2022 DiPrete BL et al. *JAMA Network Open.*

eTable 1. *ICD-9-CM* and *ICD-10-CM* Diagnostic Codes Used to Identify Opioid Overdose and Opioid Use Disorder (OUD) in Claims Data

	ICD-9-CM	ICD-10-CM
**Opioid Overdose**		
	965.00	T40.1X1A, T40.1X4A
	965.01	T40.0X1A, T40.0X4A
	965.02	T40.2X1A, T40.2X4A
	965.09	T40.3X1A, T40.3X4A
	E850.0	T40.4X1A, T40.4X4A
	E850.1	T40.601A, T40.604A
	E850.2	T40.691A, T40.694A
**Opioid Use Disorder (OUD)**		
	304.00	F11.10
	304.01	F11.120, F11.121, F11.122, F11.129
	304.02	F11.14
	304.03	F11.150, F11.151, F11.159
	304.70	F11.181, F11.182, F11.188
	304.71	F11.19
	304.72	F11.20
	304.73	F11.220, F11.221, F11.222, F11.229
	305.50	F11.23
	305.51	F11.24
	305.52	F11.250, F11.251, F11.259
	305.53	F11.281, F11.282, F11.288
	970.10	F11.29
	E9350	F11.90
	E9351	F11.920, F11.921, F11.922, F11.929
	E9352	F11.93
	E9401	F11.94
		F11.950, F11.951, F11.959
		F11.981, F11.982, F11.988
		F11.99
		T40.0X1D, T40.0X2D, T40.0X3D, T40.0X4D
		T40.1X1D, T40.1X2D, T40.1X3D, T40.1X4D
		T40.2X1D, T40.2X2D, T40.2X3D, T40.2X4D
		T40.3X1D, T40.3X2D, T40.3X3D, T40.3X4D
		T40.4X1D, T40.4X2D, T40.4X3D, T40.4X4D
		T40.601D, T40.602D, T40.603D, T40.604D
		T40.691D, T40.692D, T40.693D, T40.694D
		T40.0X5A, T40.0X5D
		T40.2X5A, T40.2X5D
		T40.3X5A, T40.3X5D
		T40.4X5A, T40.4X5D
		T40.605A, T40.605D, T40.695A, T40.695D

© 2022 DiPrete BL et al. *JAMA Network Open.*

eTable 2. Algorithm Used to Classify Monthly Prescription Trajectories

Dose in previous month	Change in dose from previous month to current month	Change in dose relative to 6-month rolling average	Classification
**>60 MME**	>10% increase from last month	>10% increase from 6-month rolling average	Increase
	>10% increase from last month	<=10% increase from 6-month rolling average	No change
	<=10% increase or decrease from last month	-	No change
	10.01-34% reduction from last month	>10% reduction from 6-month rolling average	Gradual decrease
	10.01-34% reduction from last month	<=10% reduction from 6-month rolling average (including increase)	No change
	35-99% reduction	>35% reduction from 6-month rolling average	Rapid decrease
	35-99% reduction	10.01-34% decrease from 6-month rolling average	Gradual decrease
	35-99% reduction	<=10% reduction from 6-month rolling average (including increase)	No change
	0 MME		Rapid stop
**1-60 MME**	>=10 MME increase	>=10 MME increase	Increase
	>=10 MME increase	<10 MME increase	No change
	<10 MME change	-	No change
	10-30 MME reduction but not stopped	>=10 MME reduction	Gradual decrease
	10-30 MME reduction but not stopped	<10 MME reduction	No change
	31-59 MME reduction but not stopped	31-59 MME reduction	Rapid decrease
	31-59 MME reduction but not stopped	10-30 MME reduction	Gradual decrease
	31-59 MME reduction but not stopped	<10 MME reduction	No change
**31-60 MME**	0 MME		Rapid stop
**1-30 MME**	0 MME		Gradual stop

© 2022 DiPrete BL et al. *JAMA Network Open.*

eTable 3. *ICD-10* Codes Used to Identify Fatal Opioid Overdose in Linked Death Records

Underlying Cause[a]		Contributing Cause
X40		T40.0
X41		T40.1
X42		T40.2
X43		T40.3
X44		T40.4
X60		T40.6
X61		
X62		
X63		
X64		
X85		
Y10		
Y11		
Y12		
Y13		
Y14		

[a]The death must have a both an underlying cause code and contributing cause code.

© 2022 DiPrete BL et al. *JAMA Network Open.*

eTable 4. R Packages Used in Analyses

**Package**	**Version**
broom	0.5.5
cobalt	4.1.0
cowplot	1.0.0
dplyr	0.8.5
forcats	0.5.1
ggplot2	3.3.0
ggpubr	0.2.5
ggsci	2.9
ggstance	0.3.5
glue	1.4.1
haven	2.2.0
here	0.1
Hmisc	4.4-0
knitr	1.29
lattice	0.20-38
lubridate	1.7.10
magrittr	1.5
MASS	7.3-54
nnet	7.3-12
rlang	0.4.11
rms	5.1-4
stringr	1.4.0
survival	3.2-13
survminer	0.4.6
tableone	0.11.1
tibble	2.1.3
tidyr	1.0.2

© 2022 DiPrete BL et al. *JAMA Network Open.*

eTable 5. IPTCW Risk Differences (95% CI) for the Association Between Rapid Tapering or Discontinuation of Opioid Treatment and Outcomes at Multiple Points of Follow-up

	**Fatal opioid overdose**	**Nonfatal opioid overdose**	**Fatal or nonfatal overdose**	**OUD**
3 months	0.10% (-0.06, 0.27)	0.15% (-0.09, 0.38)	0.25% (-0.04, 0.54)	0.20% (-0.65, 1.06)
6 months	0.12% (-0.06, 0.30)	0.05% (-0.20, 0.30)	0.17% (-0.14, 0.48)	0.20% (-0.79, 1.20)
1 year	0.13% (-0.09, 0.35)	0.18% (-0.13, 0.49)	0.33% (-0.04, 0.71)	0.53% (-0.65, 1.71)
2 years	0.14% (-0.12, 0.40)	0.40% (0.00, 0.79)	0.58% (0.11, 1.04)	0.47% (-1.08, 2.03)
4 years	0.38% (-0.01, 0.77)	1.04% (0.50, 1.57)	1.44% (0.78, 2.09)	2.62% (0.21, 5.04)

Abbreviations: IPTCW – inverse probability of treatment and censoring-weighted; OUD – opioid use disorder; HDLTOT – high-dose long-term opioid therapy

eTable 6. Unweighted and IPTC-Weighted Hazard Ratios (HR) for Outcomes Comparing Patients Exposed to Rapid Tapering or Discontinuation vs Patients Who Had Their Dosage Maintained or Gradually Tapered or Discontinued

	**Unweighted HR**[a] (95% CI)	**Weighted HR**[a] (95% CI)
**Fatal opioid overdose**		
0 – 12 months of follow-up	1.59 (0.68 -3.70)	1.79 (0.69, 4.69)
13 – 48 months of follow-up	1.39 (0.65 -2.99)	1.55 (0.68, 3.56)
**Nonfatal opioid overdose**		
0 – 12 months of follow-up	1.60 (1.03 -2.49)	1.26 (0.80, 2.00)
13 –48 months of follow-up	1.85 (1.22 -2.82)	2.01 (1.30, 3.12)
**Fatal or nonfatal overdose**		
0 – 12 months of follow-up	1.67 (1.12 -2.47)	1.43 (0.94, 2.18)
13 – 48 months of follow-up	1.81 (1.24 -2.64)	1.95 (1.31, 2.90)
**Opioid use disorder**		
0 – 12 months of follow-up	1.08 (0.96 - 1.21)	1.07 (0.94, 1.21)
13 – 24 months of follow-up	1.02 (0.88 - 1.19)	1.01 (0.85, 1.19)
25 - 48 months of follow-up	1.25 (1.04 - 1.51)	1.28 (1.01, 1.63)

[a]referent level is maintained dose, increased dose, or gradually decreased/discontinued dose
Abbreviations: IPTCW – inverse probability of treatment and censoring-weighted; OUD – opioid use disorder; HDLTOT – high-dose long-term opioid therapy

eTable 7. Unweighted and IPTC-Weighted Hazard Ratios (HR) for Outcomes Comparing Patients Exposed to Rapid Reduction or Discontinuation or Exposed to Gradual Reduction or Discontinuation vs Patients Who Had Their Dosage Maintained or Gradually Tapered or Discontinued

	**Unweighted HR** (95% CI)		**Weighted HR** (95% CI)	
	Gradual reduction or discontinuation[a]	Rapid reduction or discontinuation[a]	Gradual reduction or discontinuation[a]	Rapid reduction or discontinuation[a]
**Fatal opioid overdose**				
0 – 12 months of follow-up	1.02 (0.32 -3.24)	1.60 (0.63 -4.05)	0.88 (0.26, 2.93)	1.63 (0.56, 4.72)
13 – 48 months of follow-up	1.08 (0.29 -3.97)	1.46 (0.51 -4.12)	1.09 (0.27, 4.30)	1.52 (0.50, 4.62)
**Nonfatal opioid overdose**				
0 – 12 months of follow-up	1.38 (0.78 -2.46)	1.75 (1.10 -2.79)	1.24 (0.69, 2.21)	1.34 (0.84, 2.14)
13 –48 months of follow-up	1.70 (0.78 -3.69)	2.55 (1.31 -4.96)	2.24 (0.99, 5.05)	3.29 (1.64, 6.62)
**Fatal or nonfatal overdose**				
0 – 12 months of follow-up	1.26 (0.74 -2.15)	1.78 (1.17 -2.70)	1.12 (0.65, 1.92)	1.45 (0.94, 2.25)
13 – 48 months of follow-up	1.78 (0.88 -3.57)	2.57 (1.41 -4.69)	2.12 (1.00, 4.50)	3.05 (1.59, 5.85)
**Opioid use disorder**				
0 – 12 months of follow-up	1.07 (0.92 - 1.24)	1.09 (0.97 - 1.24)	1.05 (0.89, 1.22)	1.06 (0.92, 1.21)
13 – 24 months of follow-up	1.11 (0.92 - 1.34)	1.14 (0.97 - 1.33)	1.16 (0.90, 1.49)	1.05 (0.85, 1.29)
25 - 48 months of follow-up	1.73 (0.91 - 3.32)	2.00 (1.11 - 3.59)	1.30 (0.84, 2.01)	1.52 (1.03, 2.26)

[a]referent level is maintained dose or increased dose

Abbreviations: IPTCW – inverse probability of treatment and censoring-weighted; OUD – opioid use disorder; HDLTOT – high-dose long-term opioid therapy

© 2022 DiPrete BL et al. *JAMA Network Open.*

eTable 8. Outcome Frequency Overall and by Exposure Status Among a Subsample of 12 364 Patients Determined to Have Stable Baseline Dosing

	**Overall**		**Maintained, increased, or gradually reduced/discontinued**		**Rapidly reduced or discontinued**	
	**Events**	**Person-months of follow-up**[b]	**Events**	**Person-months of follow-up**	**Events**	**Person-months of follow-up**
**Fatal opioid overdose**	26	270,645	14	149,836	12	120,808
0 – 12 months of follow-up	14	118,464	9	89,619	5	28,845
13 – 48 months of follow-up	12	152,180	5	60,217	7	91,963
**Nonfatal opioid overdose**	88	269,371	39	149,611	49	119,759
0 – 12 months of follow-up	41	118,256	30	89,528	11	28,728
13 – 48 months of follow-up	47	151,114	9	60,083	38	91,031
**Fatal or nonfatal overdose**[a]	113	269,371	52	149,611	61	119,759
0 – 12 months of follow-up	54	118,256	38	89,528	16	28,728
13 – 48 months of follow-up	59	151,114	14	60,083	45	91,031
**Opioid use disorder**	1501	248,404	912	143,295	589	105,109
0 – 12 months of follow-up	816	114,139	620	87,317	196	26,821
13 – 24 months of follow-up	394	65,542	195	32,811	199	32,730
25 – 48 months of follow-up	291	68,723	97	23,165	194	45,557

Abbreviations: HDLTOT – high-dose long-term opioid therapy

[a]Some individuals had both a nonfatal and then a fatal overdose, explaining why the number of combined events is less than the number of fatal overdoses plus the number of nonfatal overdoses.

[b]Person-months of follow-up differ across each outcome analysis because an individual may have experienced a nonfatal outcome (e.g., OUD or nonfatal opioid overdose) prior to a fatal overdose. Therefore, that individual would contribute fewer person-months to the analysis with the nonfatal outcome than to the fatal opioid overdose outcome analysis.

© 2022 DiPrete BL et al. *JAMA Network Open.*

eTable 9. IPTCW Risk Differences (95% CI) for the Association Between Rapid Tapering or Discontinuation of Opioid Treatment and Outcomes Among a Subsample of 12 364 Patients Determined to Have Stable Baseline Dosing at Multiple Points of Follow-up

	**Fatal opioid overdose**	**Nonfatal opioid overdose**	**Fatal or nonfatal overdose**	**OUD**
3 months	0.10% (-0.07, 0.27)	-0.02% (-0.19, 0.14)	0.08% (-0.16, 0.31)	0.28% (-0.80, 1.37)
6 months	0.09% (-0.08, 0.26)	-0.07% (-0.27, 0.12)	0.02% (-0.24, 0.28)	0.44% (-0.89, 1.77)
1 year	0.06% (-0.18, 0.30)	0.03% (-0.28, 0.34)	0.11% (-0.29, 0.50)	0.68% (-0.89, 2.25)
2 years	0.00% (-0.27, 0.26)	0.46% (0.03, 0.88)	0.47% (-0.03, 0.96)	1.00% (-1.01. 3.01)
4 years	0.19 (-0.30, 0.67)	0.91% (0.32, 1.50)	1.11% (0.35, 1.87)	2.13% (-0.77, 5.03)

Abbreviations: IPTCW – inverse probability of treatment and censoring-weighted; OUD – opioid use disorder; HDLTOT – high-dose long-term opioid therapy

eTable 10. Unweighted and IPTC-Weighted Hazard Ratios (HR) for Outcomes Comparing Patients Exposed to Rapid Tapering or Discontinuation vs Patients Who Had Their Dosage Maintained or Increased or Gradually Tapered or Discontinued Among a Subsample of 12 364 Patients Determined to Have Stable Baseline Dosing

	**Unweighted HR[a]** (95% CI)	**Weighted HR[a]** (95% CI)
**Fatal opioid overdose**		
0 – 12 months of follow-up	1.34 (0.38 -4.77)	1.14 (0.31 -4.14)
13 – 48 months of follow-up	0.98 (0.34 -2.78)	0.98 (0.31 -3.10)
**Nonfatal opioid overdose**		
0 – 12 months of follow-up	1.18 (0.58 -2.41)	1.13 (0.55 -2.32)
13 –48 months of follow-up	2.96 (1.43 -6.13)	3.16 (1.49 -6.73)
**Fatal or nonfatal overdose**		
0 – 12 months of follow-up	1.27 (0.68 -2.37)	1.17 (0.62 -2.20)
13 – 48 months of follow-up	2.26 (1.25 -4.07)	2.28 (1.22 -4.28)
**Opioid use disorder**		
0 – 12 months of follow-up	1.09 (0.92 - 1.29)	1.09 (0.91 - 1.30)
13 – 24 months of follow-up	1.06 (0.87 - 1.29)	1.06 (0.86 - 1.32)
25 - 48 months of follow-up	1.03 (0.81 - 1.32)	1.14 (0.86 - 1.50)

[a]referent level is maintained dose, increased dose, or gradually decreased/discontinued dose
Abbreviations: IPTCW – inverse probability of treatment and censoring-weighted; OUD – opioid use disorder; HDLTOT – high-dose long-term opioid therapy

© 2022 DiPrete BL et al. *JAMA Network Open.*

eTable 11. Outcome Frequency Overall and by Exposure Status, Considering Only Nonfatal Opioid Overdoses Occurring During an Emergency Department or Inpatient Event

	Overall		Maintained, increased, or gradually reduced/discontinued		Rapidly reduced or discontinued	
	Events	Person-months of follow-up[b]	Events	Person-months of follow-up	Events	Person-months of follow-up
**Nonfatal opioid overdose**	206	472,752	89	244,135	117	228,617
0 – 12 months of follow-up	95	204,947	62	148,189	33	56,758
13 –48 months of follow-up	111	267,804	27	95,945	84	171,859
**Fatal or nonfatal overdose[a]**	259	472,752	111	244,135	148	228,617
0 – 12 months of follow-up	122	204,947	77	148,189	45	56,758
13 – 48 months of follow-up	137	267,804	34	95,945	103	171,859

Abbreviations: HDLTOT – high-dose long-term opioid therapy

[a]Some individuals had both a nonfatal and then a fatal overdose, explaining why the number of combined events is less than the number of fatal overdoses plus the number of nonfatal overdoses.

[b]Person-months of follow-up differ across each outcome analysis because an individual may have experienced a nonfatal outcome (e.g., OUD or nonfatal opioid overdose) prior to a fatal overdose. Therefore, that individual would contribute fewer person-months to the analysis with the nonfatal outcome than to the fatal opioid overdose outcome analysis.

© 2022 DiPrete BL et al. *JAMA Network Open.*

eTable 12. IPTCW Risk Differences (95% CI) for the Association Between Rapid Tapering or Discontinuation of Opioid Treatment and Overdose Outcomes, Considering Only Nonfatal Opioid Overdoses Occurring During an Emergency Department or Inpatient Event

	**Nonfatal opioid overdose**	**Fatal or nonfatal overdose**
3 months	0.15% (-0.09, 0.38)	0.25% (-0.04, 0.53)
6 months	0.04% (-0.21, 0.29)	0.16% (-0.15, 0.46)
1 year	0.16% (-0.14, 0.46)	0.31% (-0.06, 0.68)
2 years	0.36% (-0.03 - 0.75)	0.54% (0.08, 1.00)
4 years	1.03% (0.51, 1.55)	1.43% (0.79, 2.07)

Abbreviations: IPTCW – inverse probability of treatment and censoring-weighted; OUD – opioid use disorder; HDLTOT – high-dose long-term opioid therapy

eTable 13. Unweighted and IPTC-Weighted Hazard Ratios (HR) for Overdose Outcomes Comparing Patients Exposed to Rapid Tapering or Discontinuation vs Patients Who Had Their Dosage Maintained or Gradually Tapered or Discontinued, Considering Only Nonfatal Opioid Overdoses Occurring During an Emergency Department or Inpatient Event

	**Unweighted HR**[a] (95% CI)	**Weighted HR**[a] (95% CI)
**Nonfatal opioid overdose**		
0 – 12 months of follow-up	1.55 (0.98 -2.44)	1.22 (0.76 -1.95)
13 –48 months of follow-up	1.91 (1.24 -2.94)	2.10 (1.33 -3.29)
**Fatal or nonfatal overdose**		
0 – 12 months of follow-up	1.63 (1.09 -2.43)	1.40 (0.91 -2.16)
13 – 48 months of follow-up	1.85 (1.26 -2.72)	2.00 (1.33 -3.02)

[a]referent level is maintained dose, increased dose, or gradually decreased/discontinued dose
Abbreviations: IPTCW – inverse probability of treatment and censoring-weighted; OUD – opioid use disorder; HDLTOT – high-dose long-term opioid therapy

© 2022 DiPrete BL et al. *JAMA Network Open.*

eFigure 1. Death Match Algorithm

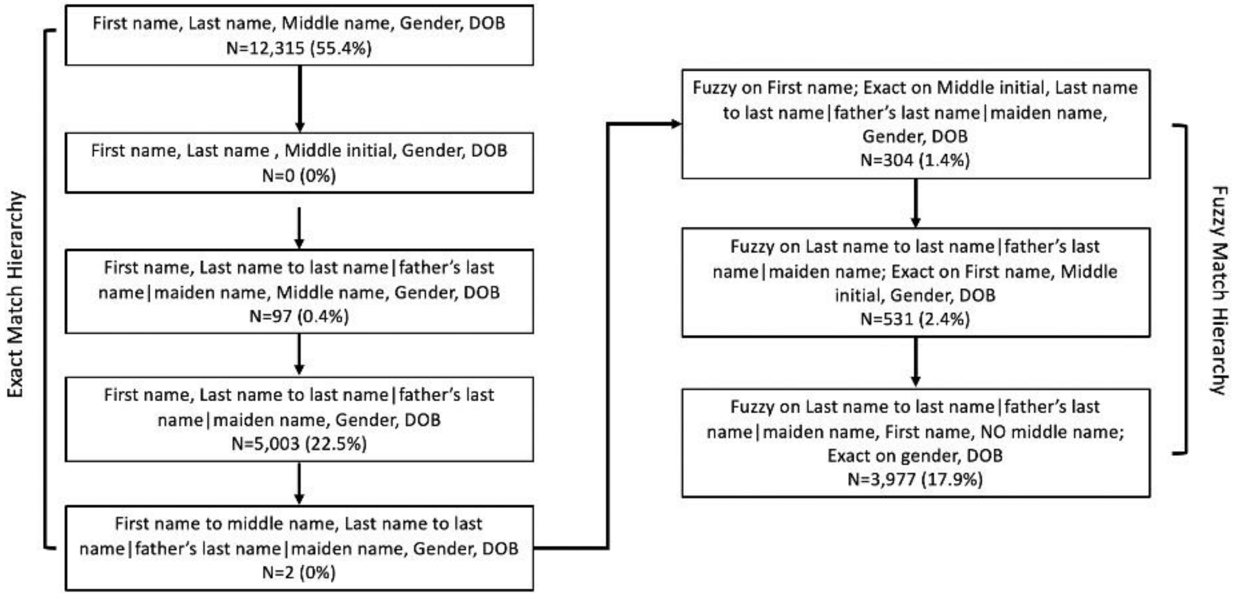

© 2022 DiPrete BL et al. *JAMA Network Open.*

eFigure 2. Timelines of Eligibility and Exposure Ascertainment and Covariate and Outcome Ascertainment

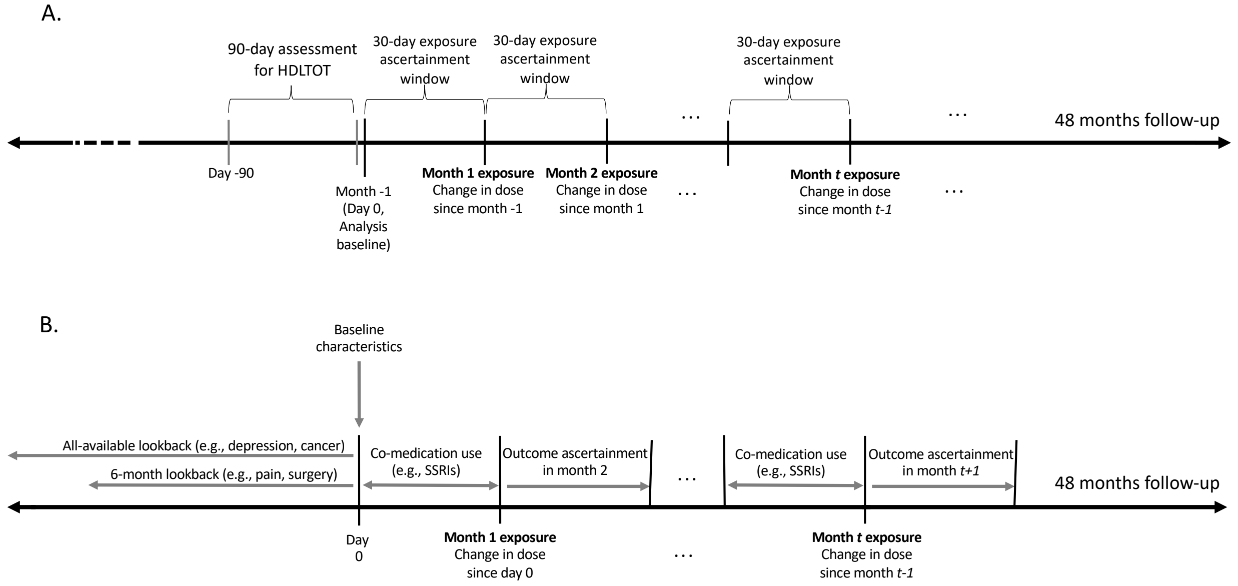

Abbreviations: HDLTOT – high-dose long-term opioid therapy; SSRI – selective serotonin reuptake inhibitor

© 2022 DiPrete BL et al. *JAMA Network Open.*

eFigure 3. Crude Cumulative Incidence of Outcomes by Primary Exposure Status

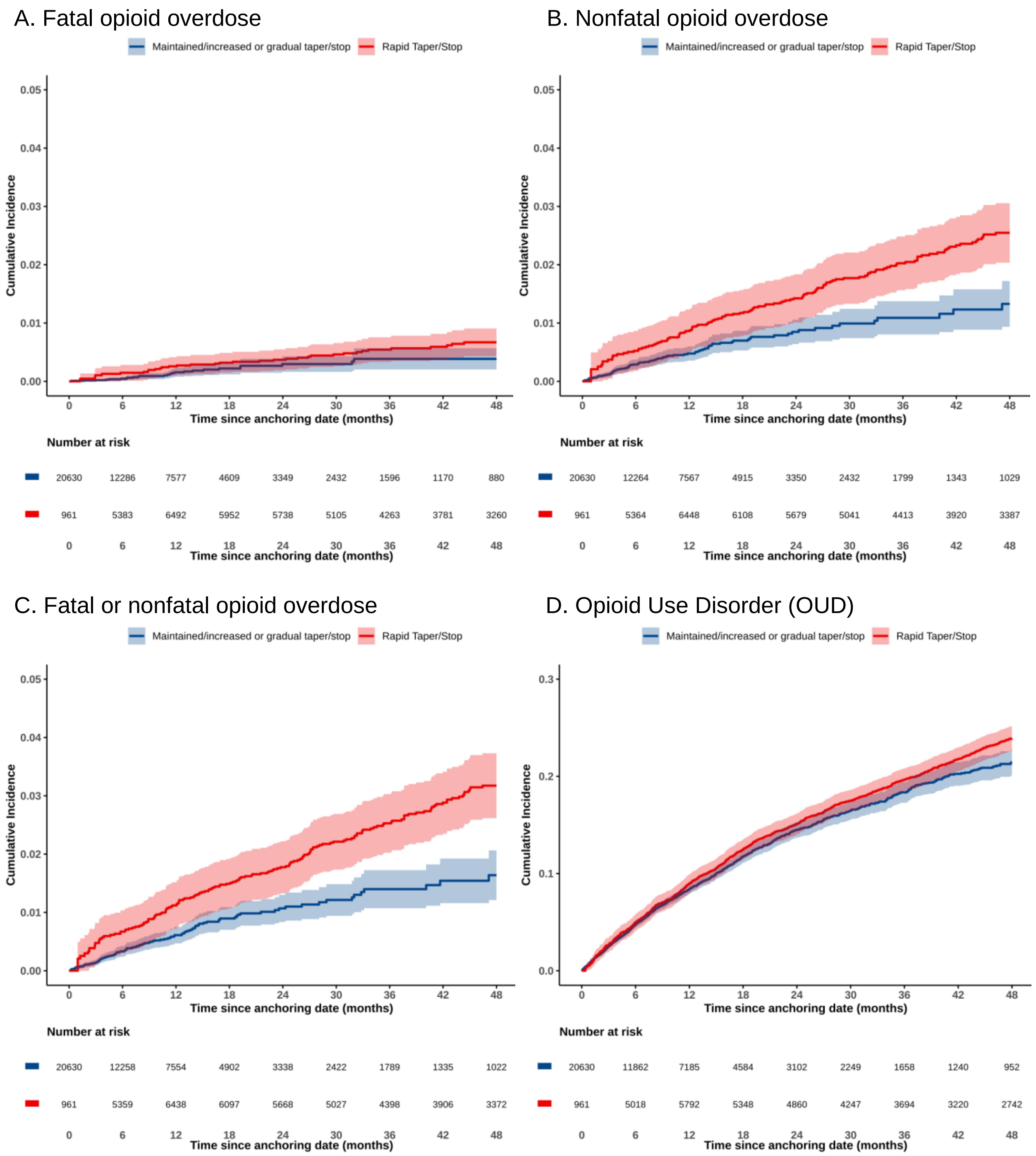

Abbreviations: OUD – opioid use disorder; HDLTOT – high-dose long-term opioid therapy

© 2022 DiPrete BL et al. *JAMA Network Open.*

eFigure 4. Crude Cumulative Incidence of Outcomes by Exposure Status Using a Categorical Exposure Definition

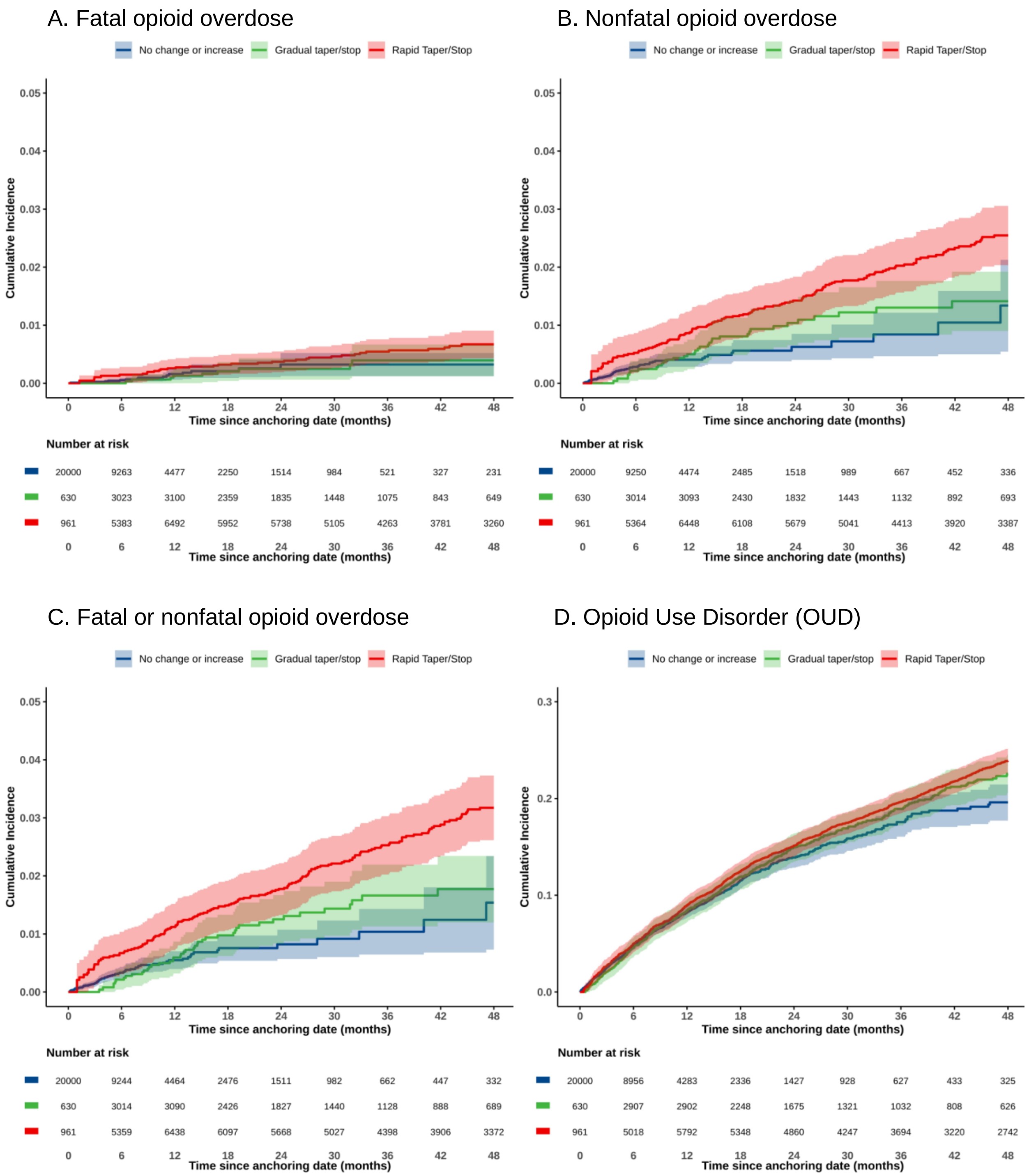

Abbreviations: OUD – opioid use disorder; HDLTOT – high-dose long-term opioid therapy

© 2022 DiPrete BL et al. *JAMA Network Open.*

eReferences

1. Conover MM, Sturmer T, Poole C, et al. Classifying medical histories in US Medicare beneficiaries using fixed vs all-available look-back approaches. *Pharmacoepidemiol Drug Saf.* 2018;27(7):771-780.
2. Brunelli SM, Gagne JJ, Huybrechts KF, et al. Estimation using all available covariate information versus a fixed look-back window for dichotomous covariates. *Pharmacoepidemiol Drug Saf.* 2013;22(5):542-550.
3. Centers for Disease Control and Prevention (CDC). *National Center for Injury Prevention and Control. Opioid Overdose: Data Resources. Analyzing prescription data and morphine milligram equivalents (MME).* 2021.
4. Ray GT, Bahorik AL, VanVeldhuisen PC, Weisner CM, Rubinstein AL, Campbell CI. Prescription Opioid Registry Protocol in an Integrated Health System. *Am J Manag Care.* 2017;23(5):e146-155.
5. Cole SR, Hernán MA. Constructing Inverse Probability Weights for Marginal Structural Models. *Am J Epidemiol.* 2008;168(6):656-664.

© 2022 DiPrete BL et al. *JAMA Network Open.*
